# Duloxetine prevents bortezomib and paclitaxel large-fiber chemotherapy-induced peripheral neuropathy (LF-CIPN) in sprague dawley rats

**DOI:** 10.1177/17448069231185694

**Published:** 2023-06-21

**Authors:** Niloufar Mansooralavi, Eugen V Khomula, Jon D Levine

**Affiliations:** Department of Oral & Maxillofacial Surgery, Division of Neuroscience, UCSF Pain and Addiction Research Center, 8785 University of California at San Francisco, San Francisco, CA, USA

**Keywords:** Chemotherapy-induced peripheral neuropathy (CIPN), large-fiber cipn, duloxetine, prevention protocol, paclitaxel, bortezomib, current perception threshold (CPT)

## Abstract

Chemotherapy-induced peripheral neuropathy (CIPN) is a debilitating, treatment-limiting, side-effect of several classes of chemotherapy drugs. While negatively impacting oncology patients’ quality of life, chemotherapy-induced large-fiber (LF) neuropathy is amongst the least well understood components of CIPN, and one for which there is currently no established therapy. Preliminary clinical observations have led to the suggestion that Duloxetine, which is used for the treatment of pain associated with small-fiber CIPN (SF-CIPN), may be effective against LF-CIPN. In the present experiments we developed a model of LF-CIPN and studied the effect of Duloxetine on LF-CIPN induced by two neurotoxic chemotherapy agents: the proteasome inhibitor, Bortezomib, a first-line treatment of multiple myeloma; and, the anti-microtubule taxane, Paclitaxel, used in the treatment of solid tumors. Since there are currently no models for selective the study of LF-CIPN, our first aim was to establish a pre-clinical model in the rat. LF-CIPN was evaluated with the Current Perception Threshold (CPT) assay, which uses a high frequency (1000 Hz) electrical stimulus protocol that selectively activates large-fiber myelinated afferents. Our second aim was to use this model to test the hypothesis that Duloxetine can prevent LF-CIPN. We report that Bortezomib and Paclitaxel induce elevation of CPT, compatible with loss of large-fiber function, which are prevented by Duloxetine. Our findings support the clinical observation that Duloxetine may be an effective treatment for the large-fiber CIPN. We also suggest that CPT could be used as a biomarker for LF-CIPN in patients receiving neurotoxic chemotherapy.

## Introduction

Chemotherapy-Induced Peripheral Neuropathy (CIPN), a form of drug-induced neurotoxicity that predominantly affects small-diameter sensory neurons, is a major dose-limiting side effect of diverse classes of cancer chemotherapy drugs.^1–4^ Many oncology patients undergoing taxane, platinum-based, and proteasome inhibitor chemotherapy report tingling, numbness, pins and needles, as well as burning, electric-like shocks, and spontaneous and evoked pain,^4,5^ compatible with small and large fiber neuropathy. CIPN symptoms vary in severity, in some patients mandating decrease in or discontinuation of treatment protocols.^6^ In cancer survivors, CIPN can persist for months or even years after completion of chemotherapy treatment, or even progress, a phenomenon referred to as “coasting”.^6–8^

Sensory neurons can be classified into fast conducting myelinated large-diameter Aβ and, thinly myelinated A-delta and small unmyelinated C-fiber (“small fiber”) Dorsal Root Ganglion (DRG) neurons, mediating predominantly non-nociceptive and nociceptive sensations.^9,10^ Small-fiber (SF) involvement of CIPN, producing pain, and large-fiber (LF) involvement attenuating touch sensation, are distinct clinical conditions that impair patients’ quality of life as nerve impairment due to toxicity depends on the class of nerve fibers effected.^11^ Although there are currently no FDA-approved treatments for either small- or large-fiber CIPN, Duloxetine is recommended by the American Society of Clinical Oncology (ASCO) for the management of the pain associated with small-fiber CIPN.^12^ In some studies of the treatment of painful CIPN with Duloxetine, it was noted that Duloxetine also appeared to improve sensory modalities associated with large-fiber function.^13–15^

Since there are currently limited pre-clinical models that allow the selective study of large-fiber neuropathy.^16,17^ to evaluate candidate treatments for CIPN we used the Current Perception Threshold (CPT) tail reflex, elicited by high frequency electrical stimulation,^18^ to evaluate changes in large-fiber function in rats treated with two neurotoxic chemotherapy agents that are known to induce LF-CIPN in oncology patients, Bortezomib^19,20^ and Paclitaxel,^13,21^ and their response to treatment with Duloxetine. The CPT test has been used successfully in preclinical studies as a noninvasive method to study large and small fiber neuropathy induced by vibration.^18,22^ A-beta fibers can be activated by 1000 Hz electrical stimulation, without activating C-fibers,^23^ which would produce pain. Therefore, in the present study we used the CPT protocol to selectively evaluate large fiber function in two preclinical models of CIPN, and their response to Duloxetine.

## Materials and methods

### Animals

Experiments were performed on male Sprague Dawley rats, with initial weight of 230–245 g, purchased from Charles River Laboratories (South San Francisco, CA). Rats were housed in the Laboratory Animal Resource Center (LARC) on the Parnassus Campus at the University of California, San Francisco. Animals were housed 3 per cage, under a 12 h light/dark cycle (cycle switch at 07:00), with controlled humidity and temperature. Food and water were available *ad libitum.*

### Current perception threshold

Rats were placed in the acrylic cylindrical restrainers that were used during Current Perception Threshold (CPT) experiments for 45–60 min and then returned to their home cage, on 3 consecutive days, to accustom them to the experimental restraint. On days when CPT was measured, animals were placed in restrainers for 30 min prior to testing, to acclimatize them to the restrainer and to attach the stimulation electrodes used for CPT testing. Animals’ tails were cleaned with Neurotron Prep Paste (GTPP 10D, Neurotron Inc., Aurora, CO), which was then wiped off with an alcohol pad. A Neurotron dispersion electrode (SDE 044, Neurotron Inc., Aurora, CO) was then placed on the base of the tail and secured by Micropore™ surgical tape. A drop of Spectra® 360 Electrode gel was applied to the Neurotron stimulating electrode (STE 0405, Neurotron Inc., Aurora, CO), which was then taped to the dorsal surface of the tail, approximately 2 cm distal to the dispersion electrode. Electrical stimuli of square wave voltage pulses, at a frequency of 1000 Hz, were generated using an S48 Grass Stimulator (Grass Instrument Co, West Warwick, RI). The stimulus intensity was increased at a constant rate (*∼0.23* *V/s*), until a response was observed. To make stimuli symmetrical around 0 the “constant” component was “filtered” by a series capacitor (47 μF). The CPT was defined as the minimum magnitude of the electrical current that produced a behavioral response consisting of a sudden, tail flick, arching movement of the tail between the electrodes, or tremor or curving of the tip of the tail. To quantify the stimulus current at CPT, a digital oscilloscope (DS1054Z, RIGOL Technologies, China) was connected across a series resistor (Rs = 390Ω) in the stimulation circuit. Once a response was observed, the peak voltage drop (V_(Peak)_) across the resistor was recorded and converted to current using Ohm's Law: *CPT=V Peak Rs*

The CPT for each animal was measured 4 times with 1min intervals between trials, and the average of the last 3 recordings used for data analysis. All CPT measurements are reported as the average of 3 trials for that day, for each rat, for 2 consecutive days. Baseline CPT for each group of rats was measured before any treatments were initiated. Since the individual operating the stimulator was not blinded to the experimental group, the CPT behaviors were video recorded to allow subsequent confirmation of responses by a blinded individual. These video recordings were randomly selected and played for the blinded researcher to score CPT responses. The concurrence rate between the 2 observers was 98%.

### Drugs

The drugs used in this study were Duloxetine, Bortezomib, and Paclitaxel. Duloxetine (TCI America, Fisher Scientific, Hampton, NH) was mixed with 5% DMSO in saline. This mixture was placed on a shaker (Eppendorf, Thermomixer R), and heated at 37°C, for 20–40 min at 750 rpm to fully dissolve. The solution was then filtered with a 0.2 µm syringe filter (Nalgene® 190-9920, Thermo Fisher Scientific, Waltham, MA) prior to intraperitoneal (i.p.) injection. Duloxetine was administered at a dose of 10 mg/kg (i.p.), once a day for 10 consecutive days. Bortezomib (LC Laboratories, Woburn, MA) was dissolved in 3% DMSO in saline and administered intravenously at a dose of 0.2 mg/kg every other day for 4 treatments. Paclitaxel (Sigma-Aldrich, St. Louis, MO) was dissolved in 15% cremophor and 15% ethanol, in saline, and administered intraperitoneally at a dose of 1 mg/kg every other day for 4 treatments.

### Duloxetine prevention protocol

Duloxetine was given daily for 10 consecutive days, starting 3 days before the start of Bortezomib or Paclitaxel treatments. The 10 consecutive days of Duloxetine treatment and 4 treatments each of Bortezomib or Paclitaxel ended on the same day. After the Duloxetine and chemotherapy treatments were completed, CPT was again measured, weekly, for 3 weeks.

### Data analysis

The effect of Bortezomib and Paclitaxel on CPT was assessed by comparing baseline CPT with CPT 1, 2, and 3 weeks after the start of chemotherapy. The effect of Duloxetine on Bortezomib- or Paclitaxel-induced changes in CPT was evaluated by comparing CPT from groups only treated with Bortezomib or Paclitaxel, in weeks 1, 2, and 3, with those treated with one of the chemotherapy drugs and Duloxetine. Data was analyzed using one-way or two-way repeated measures (RM) ANOVA, with Holm-Šídák's multiple comparisons test using Prism software, from GraphPad Software Inc. (San Diego, CA). Alpha was set as 0.05 in all statistical analyses and p-values less than that were considered significant. Animals were excluded from the study/statistical analysis if they showed signs of illness, more than 20% body mass weight loss due to chemotherapy, abnormal stress, or death.

## Results

### Duloxetine prevents Bortezomib-induced elevation of current perception threshold

**Figure 1.**
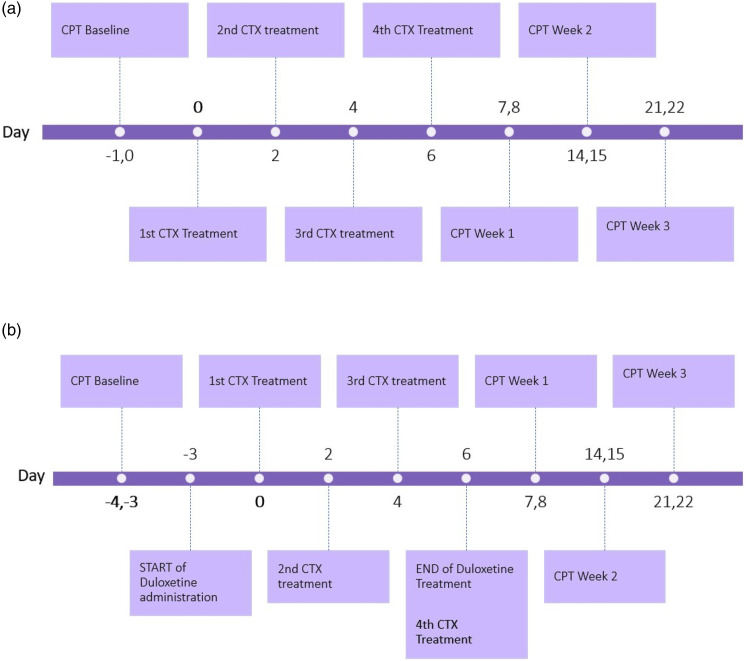
(a) Timeline for treatment with Bortezomib and Paclitaxel chemotherapy (CTX) and CPT measurements. Baseline CPT was recorded for 2 days before Bortezomib or Paclitaxel treatment was initiated. Bortezomib (0.2 mg/kg, i.v.) or Paclitaxel (1 mg/kg i.p.) was then administered intravenously (or intraperitoneally) every other day for 4 treatments. CPT was measured 24 h after the last administration of each chemotherapy drug, for 2 consecutive days, and then weekly for 3 weeks. (b) Timeline for the Prevention Protocol for the study of the effect of Duloxetine on Bortezomib- and Paclitaxel-induced large-fiber (LF) peripheral neuropathy. Two days of Baseline CPT were recorded, before the start of Duloxetine treatment. Duloxetine (10 mg/kg, i.p.) was then administered intraperitoneally every day for 10 consecutive days. Chemotherapy treatments (CTX) began 4 days after the start of Duloxetine treatments. Bortezomib (0.2 mg/kg, i.v.) or Paclitaxel (1 mg/kg, i.v.) was administered intravenously (or intraperitoneally) every other day for 4 treatments. CPT was measured 24 h after the last administration of both Duloxetine and chemotherapy drug, for 2 consecutive days, and then weekly for 3 weeks.

The timeline for the administration of chemotherapy drugs (Bortezomib or Paclitaxel) and Duloxetine, and the measurement of CPT are shown schematically in Figure 1(a)–(b).

**Figure 2.**
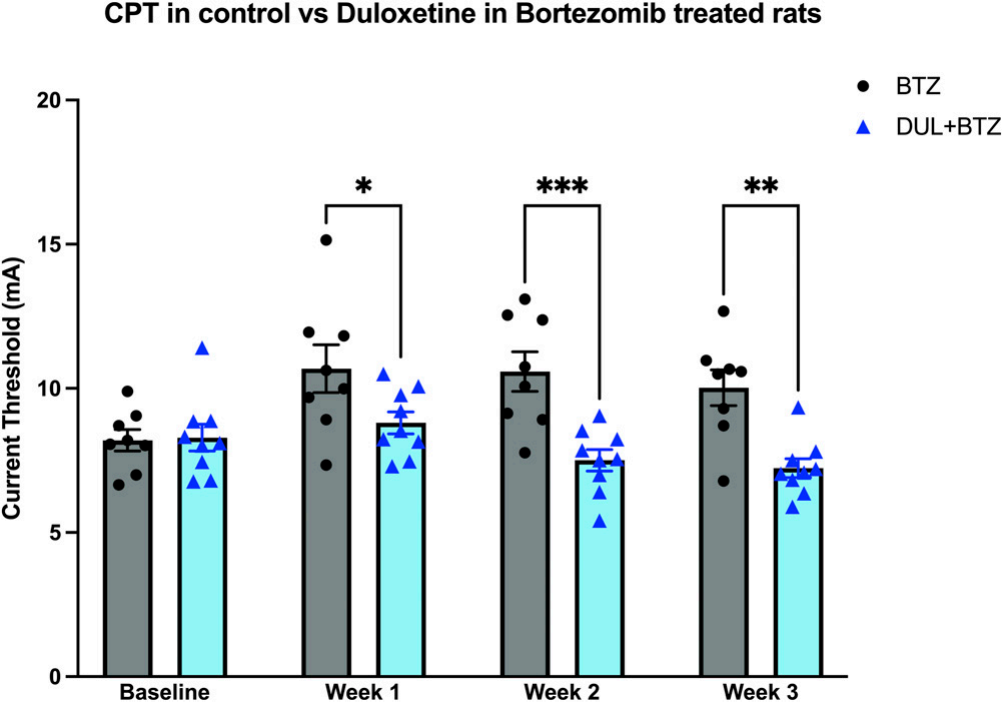
One group of rats was treated with Bortezomib, every other day for 4 treatments (BTZ); a second group was treated with Duloxetine (10 mg/kg, i.p.) daily for 10 doses, starting 3 days prior to the administration of the first dose of Bortezomib (0.2 mg/kg, i.v.) (DUL + BTZ). Compared to the BTZ baseline, CPT for the BTZ group was significantly increased after treatment (one-way RM ANOVA, F_(3, 21)_ = 4.7, *p = 0.012). CPT for the DUL + BTZ group was significantly different from that for the BTZ group (two-way RM ANOVA, Treatment Factor, F_(1, 15)_ = 16.2, **p = 0.0011; Interaction Factor F_(3, 45)_ = 4.8, **p = 0.005). Significant “time x treatment” interaction, in addition to significant effect of treatment, indicates that the difference between BTZ and DUL + BTZ groups was time-dependent and therefore cannot be just a result of vertical shift between groups (e.g., due to the difference in baselines). Pretreatment with Duloxetine significantly attenuated BTZ-induced elevation of CPT at weeks 1–3 (Holm-Šídák’s multiple comparisons test, BTZ vs DUL + BTZ: Baseline, t_(60)_ = 0.13, *p* = 0.9; Week 1, t_(60)_ = 2.5, *p = 0.03; Week 2, t_(60)_ = 4.2, ***p = 0.0004; ssWeek 3, t_(60)_ = 3.8, **p = 0.0011). Data are mean ± SEM; *n* = 8 for BTZ group and *n* = 9 for DUL + BTZ group.

Compared to baseline, Bortezomib (BTZ) induces a significant increase in CPT (Figure 2). Using a prevention protocol, in a Duloxetine-treated Bortezomib group (DUL+BTZ), rats had a significantly lower CPT, compared to animals treated only with Bortezomib (BTZ) (Figure 2). These results demonstrate that Duloxetine can prevent Bortezomib-induced elevation of CPT.

### Duloxetine prevents Paclitaxel-induced elevation of CPT

**Figure 3.**
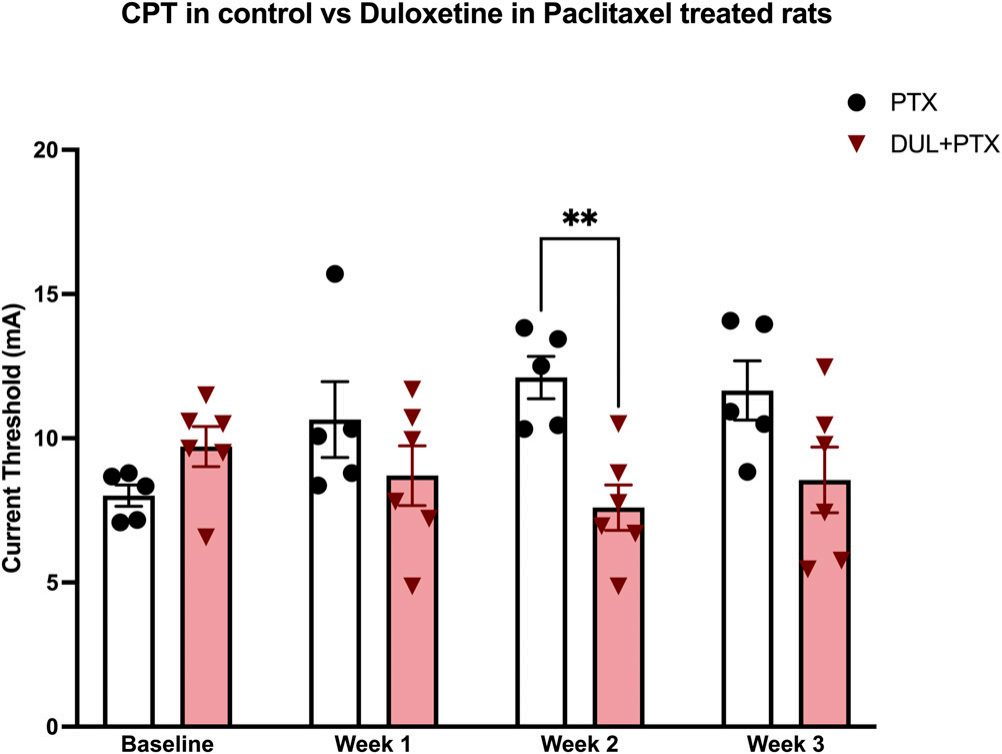
One group of rats was treated with Paclitaxel (1 mg/kg i.p.), every other day for 4 treatments (PTX); a second group was treated with Duloxetine (10 mg/kg, i.p.) daily for 10 doses starting 3 days prior to the administration of the first dose of Paclitaxel (DUL + PTX). Compared to the PTX baseline, CPT for the PTX group was significantly increased after PTX treatment (one-way RM ANOVA, F_(3, 12)_ = 5.8, *p = 0.011). CPT for the DUL + PTX group was significantly different from that for the PTX group (two-way RM ANOVA, Interaction Factor F_(3, 27)_ = 6.8, **p = 0.0015). Significant “time x treatment” interaction indicates that the difference between PTX and DUL + PTX groups was time-dependent and therefore cannot be just a result of vertical shift between groups (e.g., due to the difference in baselines). Pretreatment with Duloxetine significantly attenuated PTX-induced elevation of CPT at week 2 (Holm-Šídák’s multiple comparisons test, PTX vs DUL + PTX: Baseline, t_(36)_ = 1.3, *p* = 0.3; Week 1, t_(36)_ = 1.5, *p* = 0.3; Week 2, t_(36)_ = 3.4, **p = 0.007; Week 3, t_(36)_ = 2.3, *p* = 0.07). Data are mean ± SEM; *n* = 5 for PTX group and *n* = 6 for DUL + PTX group.

Compared to baseline, Paclitaxel (PTX) induces a significant increase in CPT (Figure 3). Using a prevention protocol, in a Duloxetine-treated Paclitaxel group (DUL+PTX), rats had a significantly lower CPT, compared to animals treated only with Paclitaxel (PTX) (Figure 3). These results demonstrate that Duloxetine can prevent Paclitaxel-induced elevation of CPT. Thus, Duloxetine reduced the severity of LF-CIPN induced by both Bortezomib and Paclitaxel.

## Discussion

Clinical evidence that Duloxetine can be used to treat large-fiber peripheral neuropathy in oncology patients with CIPN is, at present, extremely limited.^13,24,25^ In the present study, for the first time we have developed two preclinical models of LF-CIPN, one induced by Bortezomib and the other by Paclitaxel, to allow us to test the hypothesis that Duloxetine, the only drug currently recommended by the American Society of Clinical Oncology (ASCO), for the treatment of small-fiber painful CIPN, also attenuates LF-CIPN.

Since there are currently few preclinical models of LF-CIPN available,^16,17^ we first developed a model using the response to selective large-fiber activation with high frequency electrical stimulation of the rat’s tail, referred to in the literature as the Current Perception Threshold (CPT) protocol.^18^ Our results demonstrate that Bortezomib and Paclitaxel, whose anti-cancer mechanisms are quite different,^26^ but that can both cause large-fiber neuropathy in patients,^27^ produce an elevation of CPT in the rat, supporting the suggestion that Bortezomib and Paclitaxel cause loss of large-fiber function. Since the CPT protocol selectively activates large-fibers, and does not therefore produce pain, it is possible that this technique could be used as a biomarker for the study of large-fiber CIPN in oncology patients receiving neurotoxic chemotherapy drugs as well as to objectively evaluate the response of LF-CIPN to Duloxetine and potentially other treatment modalities.

Our finding that Duloxetine prevents the elevation of CPT caused by Bortezomib and Paclitaxel in the rat, supports the limited clinical observations suggesting that Duloxetine may be able to attenuate LF-CIPN,^13,25^ as well as the need for well-powered studies of the effects of Duloxetine in patients with LF-CIPN.^25,28^ Our study also provides a preclinical model that can be used to evaluate large-fiber function in rats being treated with other classes of chemotherapy drugs, and other candidate treatments for large-fiber CIPN, to precede the time consuming and costly process of evaluating the clinical efficacy and safety of drugs for the prevention and treatment of CIPN in clinical studies.^28^ Finally, since the CPT protocol only activates large-diameter myelinated sensory axons and, therefore, does not produce pain, CPT may be useful to monitor large-fiber function in patients receiving neurotoxic chemotherapy drugs, to ascertain if patients are developing LF-CIPN and if patients developing LF-CIPN are responsive to treatment.

Duloxetine, a well-characterized clinical serotonin-noradrenaline reuptake inhibitor (SNRI), is thought to produce its anti-hyperalgesic/analgesic effect, including for neuropathic pain,^29^ by acting as a neurotransmitter reuptake inhibitor, for serotonin and noradrenaline, in endogenous descending pain control circuits.^30,31^ However, how this mechanism could impact symptoms associated with LF-CIPN remains to be elucidated.

In a recent study, Duloxetine was shown to both prevent and reverse Bortezomib- and Paclitaxel-induced SF-CIPN, in the rat,^32^ an effect that is dependent on the function of the hypothalamic-pituitary-adrenal and sympathoadrenal neuroendocrine stress axes. Of note in this regard, Duloxetine is also highly effective as an anxiolytic in humans,^33^ reducing activity in the neuroendocrine stress axes.^32^ While touch, a sensation mediated by large fiber afferents, can attenuate neuroendocrine stress axis function,^34^ whether neuroendocrine stress axes influence large-fiber function, including if large-fiber CIPN is stress hormone dependent, as is small-fiber CIPN,^32,34^ remains to be determined.

Recent studies have described another effect of Duloxetine that may be relevant to its impact on CIPN in general and LF-CIPN in particular. Thus, it has been shown that Duloxetine also inhibits Organic Cation Transporter 2 (OCT2), a mechanism that is well-established to facilitate the uptake of Oxaliplatin into Dorsal Root Ganglion (DRG) neurons, thought to prevent Oxaliplatin-induced peripheral neuropathy (OIPN).^35^ While this study demonstrated that Duloxetine inhibits the uptake of Oxaliplatin into DRG neurons, Duloxetine has not been studied with reference to inhibiting transporters involved with Paclitaxel- and Bortezomib-induced peripheral neuropathy. While the transporters that facilitate the uptake of Paclitaxel into DRG neurons have been studied *in vitro* and in rodent models,^36^ this does not appear to be mediated by OCT2. And their response to Duloxetine has not been evaluated. Furthermore, knowledge of the mechanism by which Bortezomib is taken up into DRG neurons is limited.^36,37^ Thus, further research is required to establish the involvement of transporters in Bortezomib- and Paclitaxel-induced neuropathy and Duloxetine's effect on their activity. Additional studies have shown that Duloxetine and other SNRIs inhibit activation of p38 MAPK and NF-κB, which have both been suggested to contribute to neuropathic pain through a pro-inflammatory cascade.^38^ However, how this mechanism might impact LF-CIPN remains to be studied.

A limitation of the current study is that all experiments were performed in male rats. Since sex differences in CIPN and its response to treatment with Duloxetine have been described,^34,39^ it will be important to perform parallel experiments in female rats.

In conclusion, our experiments demonstrate, using CPT and established CIPN protocols in the rat, that two chemotherapy drugs, Bortezomib and Paclitaxel, induce large-fiber loss of function in the rat. Our findings support the suggestion that Duloxetine, an SNRI, can prevent the development LF-CIPN induced by neurotoxic chemotherapy drugs, in preclinical animal models, supporting to the currently limited data reported in clinical studies. Given that Duloxetine is an FDA approved drug that has been used to treat CIPN pain in patients, CPT could be used to evaluate for its effect on LF-CIPN in patients receiving neurotoxic chemotherapy drugs.
